# Combining X-ray Fluorescence and Monte Carlo Simulation Methods to Differentiate between Tumbaga and Gold-Alloy or Gildings

**DOI:** 10.3390/ma15134452

**Published:** 2022-06-24

**Authors:** Antonio Brunetti, Marta Porcaro, Angel Bustamante, Giovanni Stegel, Roberto Cesareo

**Affiliations:** 1 Biomedical Sciences Department, University of Sassari, 07100 Sassari, Italy; 2 Chemistry, Physics, Mathematics and Natural Science Department, University of Sassari, 07100 Sassari, Italy; mporcaro@uniss.it (M.P.); stegel@uniss.it (G.S.); 3 Solid State Physics Department, National University of San Marcos, Lima 15081, Peru; angelbd1@gmail.com; 4 Institute of Mathematics and Physics, University of Sassari, 07100 Sassari, Italy; roberto.cesareo@gmail.com

**Keywords:** gradient, depletion, XRF, multilayer, Tumbaga

## Abstract

Depleted metals have been produced since many centuries ago. Probably the most famous examples from ancient times are the so-called Tumbaga gold artifacts, whose production was introduced by the pre-Colombian civilizations. Tumbaga-like structures have been identified also in modern nanotechnological materials. In both cases, but specially for the ancient Tumbaga, due to their preciousness, their characterization should be obtained by non-destructive analysis. Several analytical protocols have been developed, some of them non-destructive, such as those based on X-ray Fluorescence, but the results obtained do not always allow for a reliable identification of Tumbaga with respect to gilding or single alloy samples. Besides the capability to distinguish between different structures of the sample, it is also important to obtain a quantitative estimation of its composition. In order to meet this demand, a new approach based on X-ray Fluorescence coupled to Monte Carlo simulations is proposed. It allows one to distinguish easily between the three manufacturing techniques and to quantify the composition of the sample without any destructive sampling. It constitutes a new tool for the study of complex alloy structures. The protocol is applied here to some ancient Tumbaga gold samples and is described in detail, comparing the results to those obtained with other techniques.

## 1. Introduction

**Figure 1:**
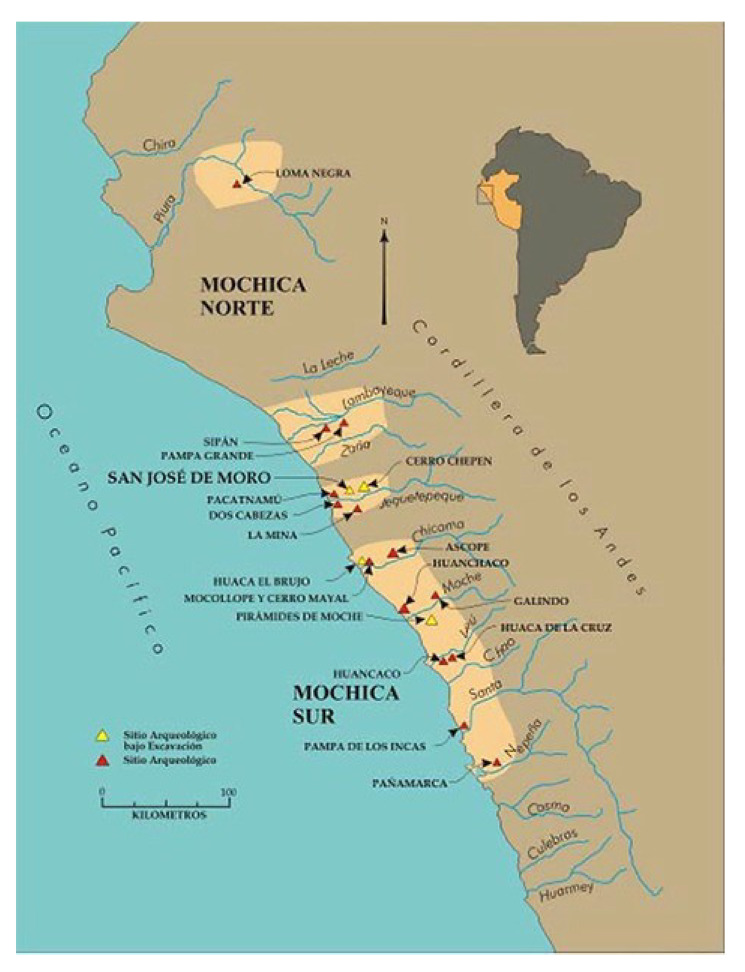
Map of current day Peru, showing the Moche pre-Colombian civilization.

The development of metallurgy in Central America produced a huge number of marvellous gold and silver artifacts and some innovative manufacturing techniques [1,2,3]. These artifacts are usually body protections or ornaments, and are based on a combination of three main metals, namely gold, silver and copper, sometimes with the addition of semi-precious stones. These gold artifacts are often related to the power of the owner or connected to religious offerings. The metallurgic technology developed has interested many generations of archaeologists and it is not completely known, as in the case of so-called Tumbaga. This name refers to Au-Ag-Cu alloys which have undergone a depletion process producing a gold (gold-Tumbaga) or silver (silver-Tumbaga) enrichment on the external surfaces. So high is the quality of this ancient process, that these artifacts appear even today as pure gold artifacts with almost no aging effect. Besides Tumbaga, also gilded copper objects as well as Au- or Ag- alloy artifacts have been discovered. Many pre-Colombian civilizations used these techniques, including the Moche (or Mochica) civilization (I-VII centuries A.D.), which is one of the most important civilizations of ancient Peru (Figure 1).

In this paper we present and discuss some of the results obtained on a set of gold objects coming from the treasure of the tomb of the “Lord of Sipan”. His burial was discovered in 1987 by the Peruvian archaeologist Walter Alva at Huaca Rajada, also known as Sipan (Lambayeque Valley, North of Peru) and is a part of a funerary platform containing several mummies [4]. It has been the first finding of a burial of a person of high rank and it has changed our knowledge about Moche civilization [4,5]. The discovered objects are now kept at the Royal Tombs of Sipan Museum (Chiclayo, Peru).

The burial contains, besides the body, hundreds of precious objects made of gold, silver and copper, and semi-precious stones, all of them confirming the importance of the Lord of Sipan. Many attempts have been made, most of them destructive, to differentiate and analytically define a Tumbaga. There is a need for a precise, stratigraphic determination of the alloy composition [6,7,8,9,10,11,12,13,14,15,16,17]. However, a destructive approach limits the number of pieces one is allowed to examine. Thus, a non-destructive approach is desirable and in this sense X-ray fluorescence (from here on XRF) is undoubtedly the most used technique in Cultural Heritage studies. Moreover, these ancient precious artefacts are immovable, and portable instruments are required. It is relatively easily to project and assemble an XRF portable system fashioned around a specific problem such as the metal composition characterization [6].

XRF is based on interaction of an X-ray photon beam with a sample. This interaction changes the incident beam producing a new set of photons (secondary photons) which contain information about the sample composition. This set of photons is usually represented by a histogram of the number of photons vs their energy, called X-ray spectrum. In general, an X-ray spectrum is composed of a set of X-ray fluorescence peaks superimposed onto a background. The position of the peaks, that is their energy, is fixed for each chemical element, so giving a marker of the presence of a specific chemical element. The peak height is roughly connected to the concentration. In general, each chemical element produces more detectable XRF peaks whose relative intensities, that is their ratios, can be theoretically determined. Sometimes, this ratio can be used to differentiate gilded copper objects from gold-alloy or Tumbaga. Let us explain this better by considering, for simplicity, two layers where the outer one is composed of gold (gilding) and the inner one of copper. The incident X-ray photon beam, when crossing the gold layer, interacts with the copper layer which emits photons at characteristic energy. The XRF copper photons, usually the so-called Kα and Kβ lines, travel back to the surface crossing the outer gold layer where they are partially absorbed. The degree of absorption depends on gold foil thickness and the different energy of Cu-K lines, in the sense that photons at higher energies (Kβ) will be less absorbed than those at lower energy (Kα), so altering their ratio.

**Figure 2:**
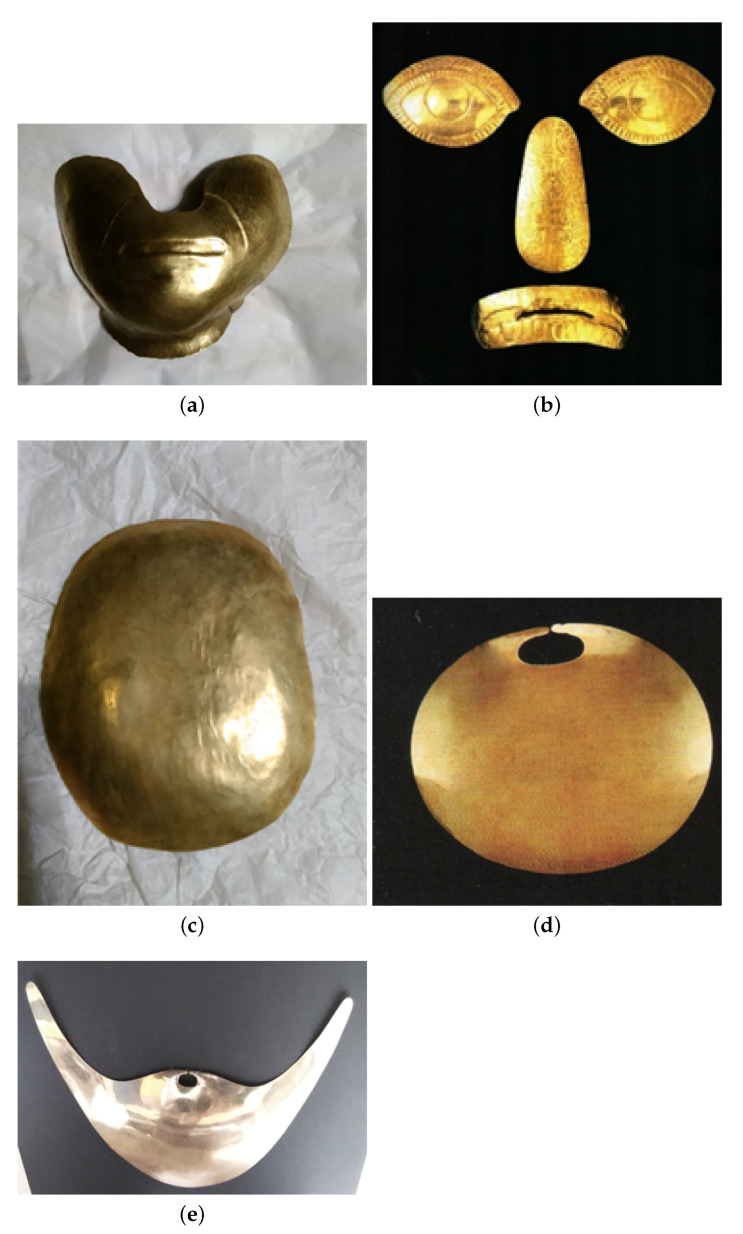
Pictures of most of the samples examined here and kept at Museum Royal Tombs of Sipan, (Lambayeque, Peru). (**a**) Chin protector; (**b**) Right and Left eye protector, Nose protection, Tooth protection; (**c**) Brain container; (**d**) Convex nose protector; (**e**) Nose decoration.

This allows one to evaluate the thickness of the outer layer, to obtain the position of the copper (first and/or second layers) and its concentration. This approach is effective when two well defined layers are present, as in the case of gilded copper, but not in the case of Tumbaga, where the relative concentrations of gold, silver and copper change continuously from the surface to the bulk. Thus, a gradient-like model is more suitable to represent the real structure. In order to solve this problem a method was applied in the past, based on the transmission of monoenergetic X or γ-rays, such as the 59.6 keV emitted by Am-241 and XRF measurements [17]. If the artifact is an Au-alloy, then the surface composition would correspond to the bulk composition. Alternatively, if the artifact is a Tumbaga, the artifact would be practically copper, with a low concentration of gold. Moreover, the amount of transmitted photons is quite different for the two types of alloys [17]. However, the limit of this method is that it requires the measurement of the sample thickness and use of photons at higher energies compared to the common XRF instruments, both of which are not always possible. A new method is therefore examined, discussed and proposed in this paper, combining XRF and Monte Carlo simulation, and in which the gradient structure is modelized by a superimposition of several, micrometric, layers. The protocol is described in detail in the next section. However, it can be useful to say a few words about Monte Carlo methods, leaving an in-depth and complete description to ref. [18]. A Monte Carlo simulation (from here on MCS) is a probabilistic technique normally used to study high-dimensional problems which cannot be tackled by usual analytical methods [19]. In the case under consideration, the MCS reproduces the real experimental results by modelling geometry of the experimental system, X-ray emission, detector response as well as sample composition and structure. Several artifacts from the tomb of the Lord of Sipan (see Table 1 and Figure 2) were tested using this new approach, denomined from here on XRF-MCS).

## 2. Materials and Methods

**Figure 3:**
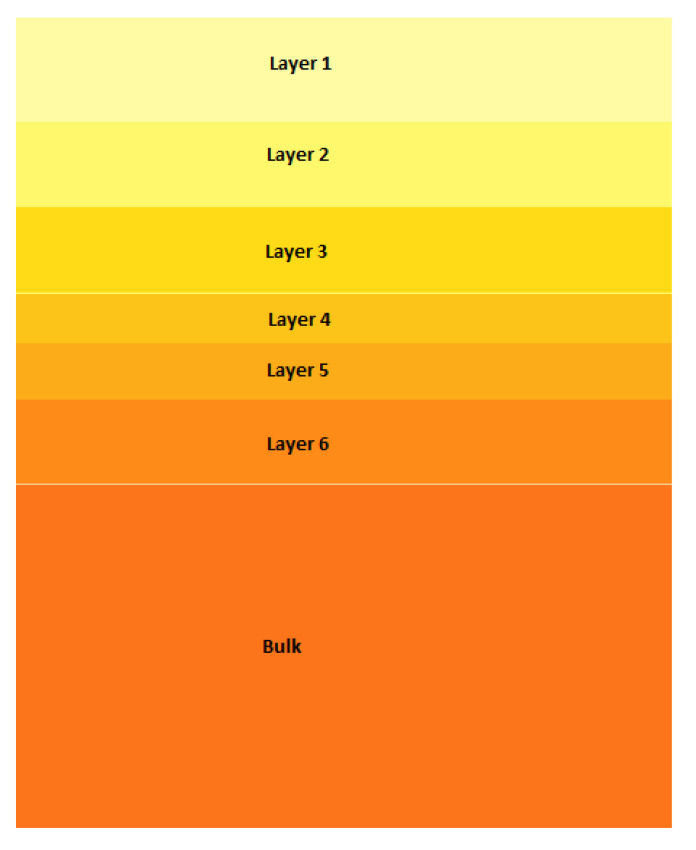
Schematization of a seven-layer structure used for Monte Carlo simulations.

Monte Carlo Simulation represents a virtual reproduction of a real experiment. Thus, each part of the experimental apparatus must be accurately modelled: the X-ray emission, coming from the X-ray tube, and the response of the detector. Besides that, the sample also must be modelled. This is the only MCS part which is continuously tuned according to the results of the simulation. The MCS protocol works as follows: a hypothetical model of the sample is chosen. Then a simulation is performed, followed by a comparison of the simulated spectrum with the measured one: if differences are observed, the model of the sample is changed and a new simulation is started. These steps are repeated until a perfect reproduction of the measure spectrum is obtained, as measured by a chi-squared test. When this happens, the model of the sample, both in structure and composition, can be regarded as a good reproduction of the real one. This protocol has been used in many published papers, mainly concerning bronzes [20,21]. The Monte Carlo software used in this paper is called XRMC and enables the production of a simulated spectrum similar, in a statistical sense, to a measured one in less than one minute [22]. It is also accurate because the atomic data required for the simulation, the Xraylib database [23], is continuously updated and tested by many researchers all over the world. In the case of a bronze, the model is usually formed from two up to three layers: patina (one or two layers) and bulk bronze. In the case of Tumbaga the model is much more complex. Both XRMC and XRaylib software have been developed at University of Sassari in collaboration with other research center such as the European Synchrotron Radiation Source (ESRF) at Grenoble, France. They can be freely downloaded [24,25]. Several models have been tested in this paper, ranging from one up to eleven layers. A sketch of such a kind of structure for a Tumbaga gold is reported in Figure 3, where each layer is formed by a different combination of gold, copper and silver (described by the change of tonality/color) and in principle by different thickness. The lower layer, denominated “bulk” has an infinite thickness (from the point of view of attenuation).

It is easily guessed that an eleven-layer model has a huge number of parameters. In fact, even in the case that each layer is composed of three elements only, copper, silver and gold, the number of parameters reaches 44! (eleven layers with three elements and eleven thicknesses each). Moreover, the three chemical elements are present in all the layers with sometimes similar concentrations, so making it harder to characterize the sample. Dealing with so many parameters, in order to avoid an ill-posed problem, that is, a problem with multiple solutions, many combinations of composition/layers number have been performed here and their influence over the simulation tested. Three classes of models were tested: Au-alloy, gilded copper and Tumbaga. As mentioned above, all of them have been applied to artifacts previously analyzed by XRF, where the differential attenuation approach [6,17] has produced doubtful results in establishing the exact topology, Tumbaga–gilded–single alloy, of the sample examined. In the case of the chin protector, a Tumbaga object (Figure 2a), the results have been compared to those reported in ref. [10] obtained with the AES techniques, so allowing us to check the method against a high-resolution, but destructive, technique. In the case of the brain container, the XRF-MCS results are compared to those obtained by XRF with γ-transmission ([17]).

**Figure 4:**
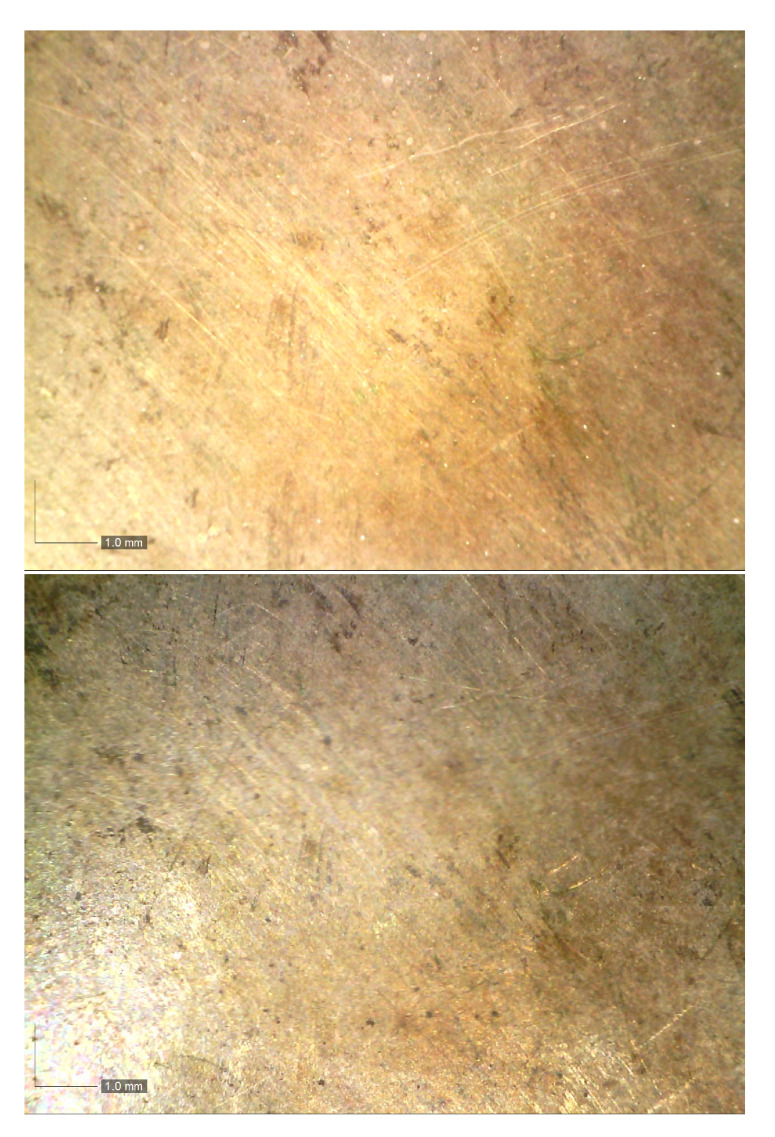
Microphotos of Tumbaga samples (30× magnification).

**Figure 5:**
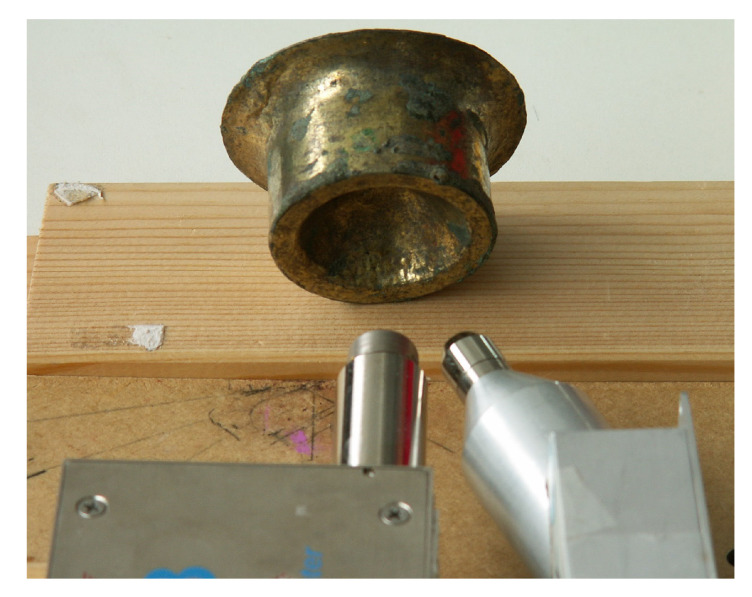
Picture of the experimental setup geometry: Uncollimated detector (on the left) placed vertically with respect to the surface of the sample and X-ray tube usually forming an angle between $30^{\circ }$ and $45^{\circ }$.

The experimental setup consists of an X-ray tube, a silver anode, operating at 40 kV, 5–15 μA, 1 mm-wide collimated, and an SDD detector without any collimation, both produced by Ametek-Amptek Inc. (Berwyn, PA, USA). The X-ray beam is unfiltered. This is an unconventional choice because, after the interaction with the sample, it will produce a large background, which is an undesired effect if the peaks area must be extracted. However, the background contains information about the so–called dark matrix, that is the part of chemical composition of the sample that does not produce any detectable fluorescence peaks. For example, this approach allows one to determine the thickness of the protective layer, usually placed on the metallic surface in order to block the corrosion process (this is not the case of the sample examined here because the external layer, essentially composed of gold, prevents large corrosion processes). Regarding the choice of the operating voltage, it is due to three principal constraints: first, obtaining a perfect reproduction of the X-ray beam emitted by the X-ray tube, which requires a very long time, second, obtaining a versatile spectrum, applied for different type of samples, in our case from bronze to gold, and lastly, the voltage being usually available for a portable, small, X-ray tube. A 40 kV voltage fits well with these constraints. Finally, it will be useful to say a few words about the choice to use a silver anode for samples containing silver, instead of an apparently more convenient and more common rhodium anode. The choice was due to the different shape of the X-ray tube emission, where, at the same operating voltage, a silver anode produces more photons at higher energies (say around 30 kV) than a rhodium one, hence a better spectrum from the statistical point of view, where the statistical noise on the counts of a specific energy channel is proportional to the square root of the number of counts itself. Regarding the geometry of the system, it can be optimized according to the sample shape. In general, the X-ray tube is placed at $45^{\circ }$, 2 cm from the sample surface, while the detector is placed vertically, 2 cm from the sample surface. This setup minimizes the effects of irregularities of the surface on the measured spectrum. Two microscopic photos acquired at 30× magnification (Dino–Lite microscope, AnMo Electronics Corporation, Hsinchu, Taiwan) of a typical Tumbaga surface are shown in Figure 4. The dark-brown spots should be an effect of corrosion of the copper which has risen to the surface and the very thin straight lines, or better the scratches, are probably due to the Tumbaga manufacturing technique. The same kind of structure is reported in [10]. However, the influence of scratches on the spectrum is, in our case, minimized, due to the size of the focal spot, about 2 mm wide, larger than the scratch width. The same does not hold for any of the techniques using the micrometric approach [10]. In Figure 5 a picture of the experimental setup is shown.

## 3. Results

**Figure 6:**
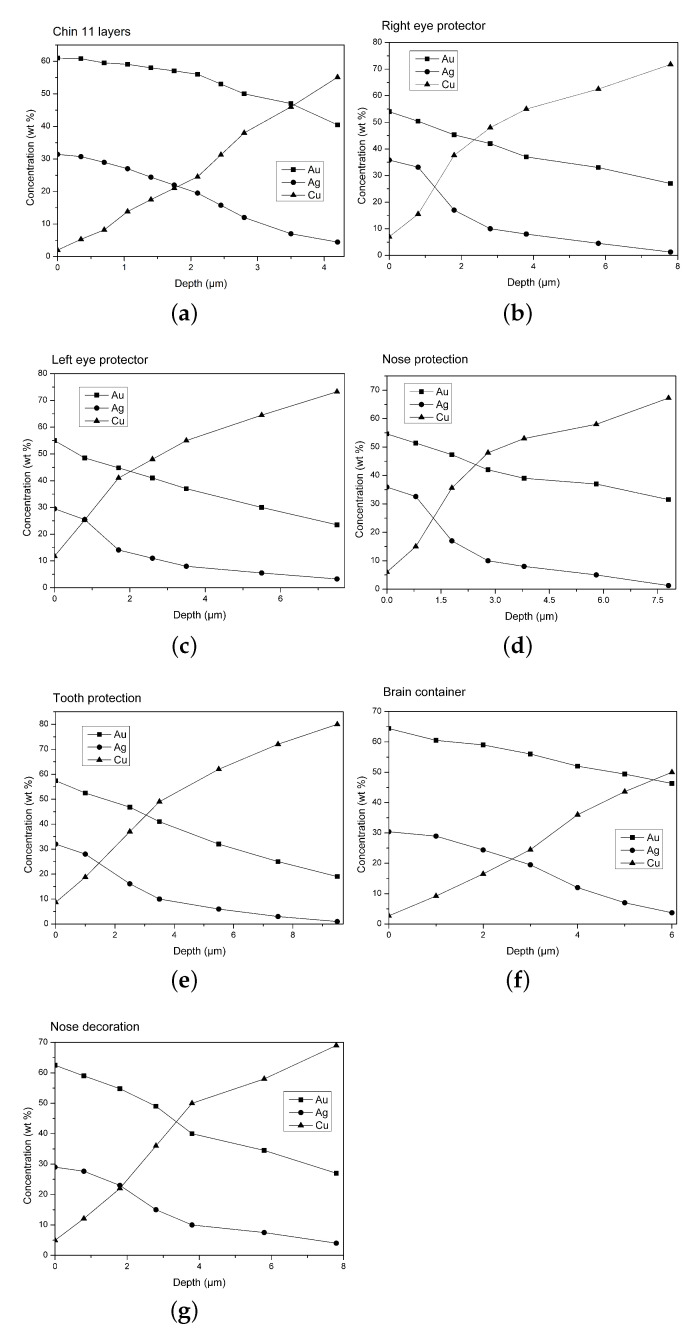
Estimated trends of gold, copper and silver concentrations vs. depth profiles for each Tumbaga sample examined. (**a**) Chin 11 layers; (**b**) Right eye protector; (**c**) Left eye protector; (**d**) Nose protector; (**e**) Tooth protector; (**f**) Brain container; (**g**) Nose decoration.

**Figure 7:**
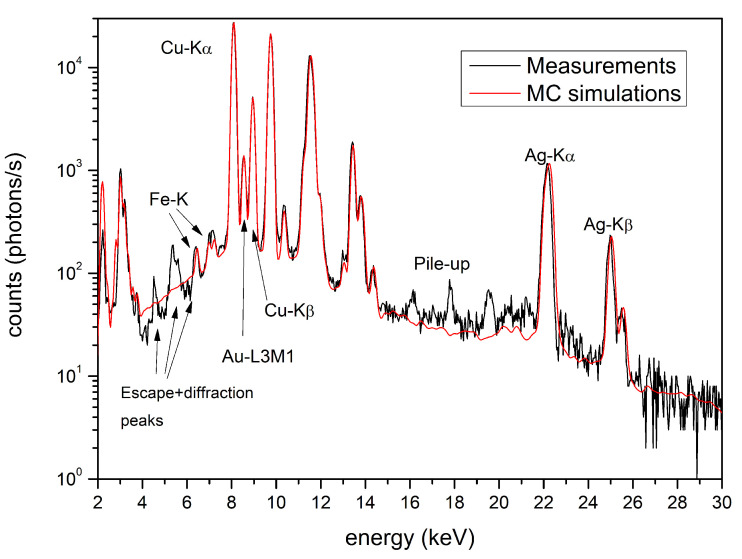
Chin protector. Comparison between MCS and XRF measured spectra. The unfitted peaks are due to pile-up, escape and diffraction phenomena and so not attributable.

A fragment of the chin protector (Figure 2a), was studied in detail by Hörz and Kallfass [10], who demonstrated that this artifact is Tumbaga. Our estimation by XRF-MCS, with the related concentration curves vs. depth, confirms this conclusion (Figure 6a and Table 2). The chin-protector composition at different depths obtained with XRF-MCS is reported in Table 2, while comparison of the simulated and measured spectra is depicted in Figure 7. The best fits of all the Tumbaga samples have been obtained with a seven-layer structure, but the chin-protector best fit has been obtained with an eleven-layers model. The gradient profile of the chin protector is similar but not equal to that in [10]. This can be due to a different point of measurement and the different sensitivity to the scratches. As for the four artifacts covering and protecting the face of the corpse of the Lord of Sipan (protectors of eyes, nose and mouth, see Figure 2b), they have been previously identified as Tumbaga from EDXRF-measurements [6,17], but only from a qualitative point of view, from their visual aspect and from their thickness, about 1 mm.

**Figure 8:**
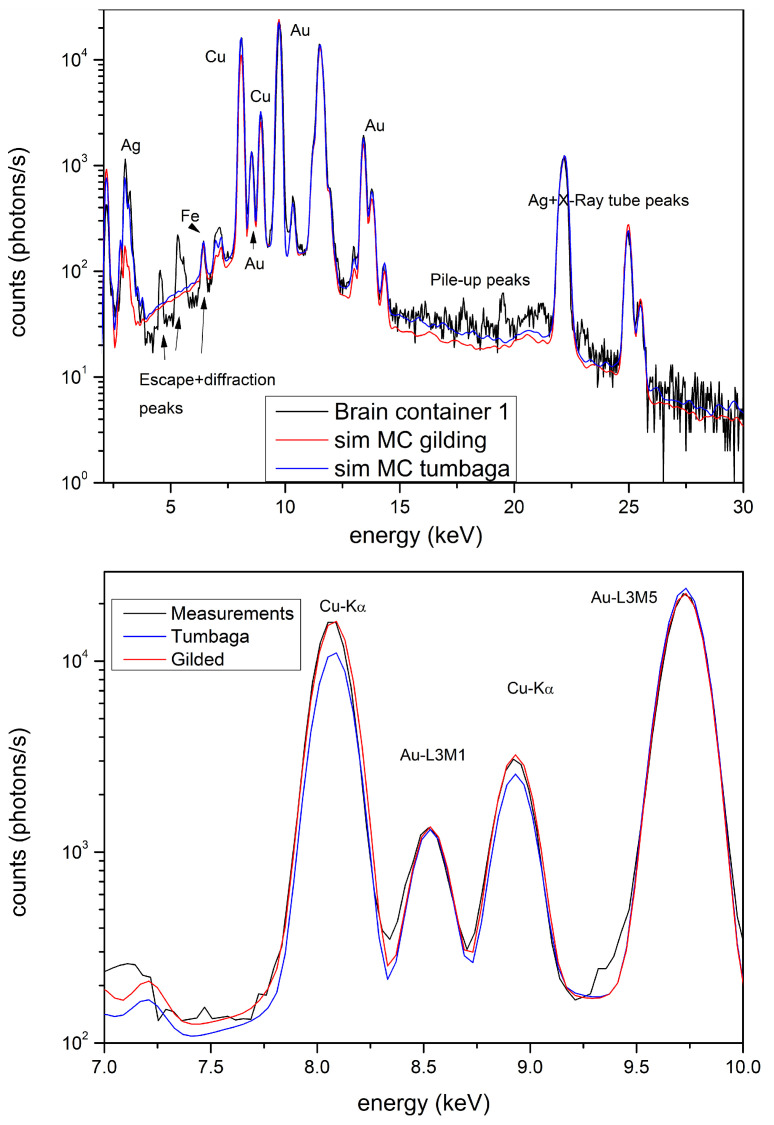
Brain protector. Comparison of the simulated X-ray spectra of gilded copper, Tumbaga and XRF measured spectra. In the figure at the bottom the peak strcture in the 7–10 keV range is depicted. It clearly shows the effect of the different models on the quality of the estimate.

The gradient profiles of these artifacts are reported in Figure 6b–e and they clearly demonstrate that these artifacts are surely Tumbaga. The brain container (Figure 2c) was previously analyzed both by EDXRF-analysis and by transmission measurements, using 59.6 keV γ-rays [17]. It was shown that this artifact is also Tumbaga. XRF-MCS confirms this result and the Au, Ag and Cu concentration profile vs. depth is shown in the same (Figure 6f). A comparison of Tumbaga and gilded-copper best simulated spectra vs measured one for this artifact is reported in Figure 8: the Tumbaga model simulation proved to be much better than the gilded one. The convex nose protector was previously analyzed by EDXRF only ([15,17]), leaving doubts about is classification (Au-alloy or Tumbaga).

**Figure 9:**
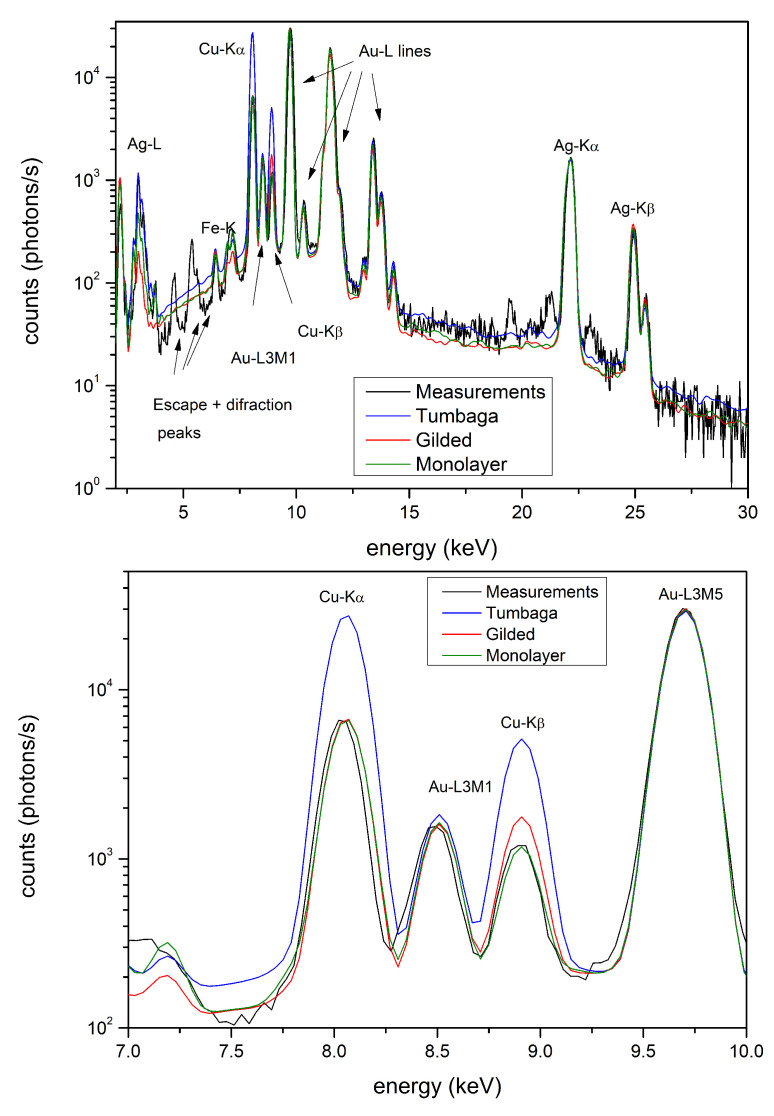
Nose protector: Comparison between the EDXRF-measured spectrum and that simulated by MCS for Tumbaga, Au alloy and gilded copper. In the figure at the bottom the peak structure in the 7–10 keV range is depicted. It is clearly visible the effect of the different model on the quality of the estimative.

The artifact was therefore accurately studied by XRF-MCS and it is incontrovertibly a gold-alloy: Au (71%), Ag (18%) and Cu (11%). In order to test the quality of this result it has been also compared with the best results obtained with a Tumbaga model as well as a gilded-copper model. The spectra relative to these simulations are reported in Figure 9. It is clear that it is impossible to obtain a good reproduction of the experimental spectrum by using a Tumbaga or a gilded-copper model (see inset in Figure 9). Finally, two other important questions must be considered:

- is there more than one optimal solution for the gradient profile?
- what is the minimum detectable change in the slope of gradient?

**Figure 10:**
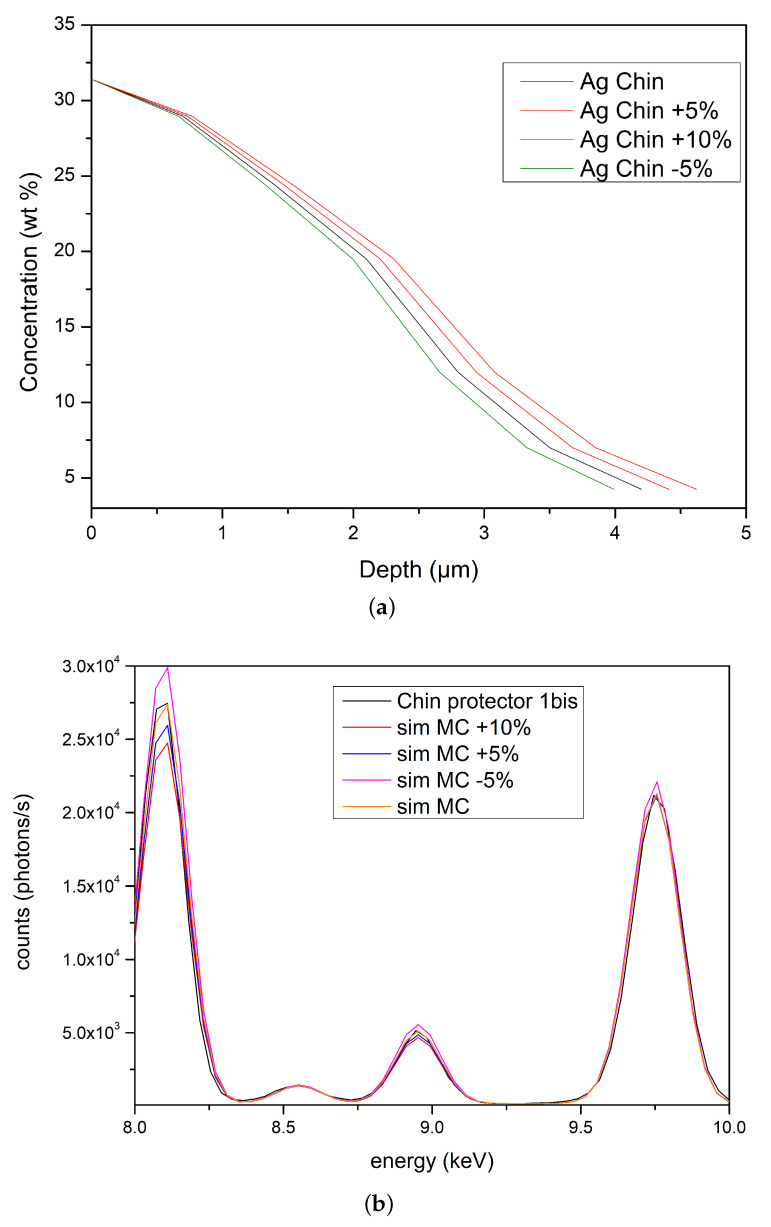
Influence of changes in the silver gradient slope on MCS simulations. (**a**) different slopes and (**b**) corresponding spectra (copper peaks detail).

In Figure 10a different gradient slopes have been simulated for the Ag concentration. The changes in the slope have been simulated by changing the thickness of all layers by a fixed percentage and adjusting the concentrations in each layer in order to optimize the fit (see Figure 10a key). As in all the other cases reported here, copper peaks are the principal index for model classification (Figure 10b). This is due to the energies of their fluorescence peaks being mostly lower than the energies of the other peaks. In fact, the lower the energies, the higher the attenuation brought about by the material crossed. Changes as low as 5% of the thickness can be easily detectable, making the method proposed highly sensitive and so reliable for estimating the gradient profile. Sensitivity to changes in concentration has been tested on the inner layer, that is in the worst condition, because sensitiveness is decreased by the attenuation of all the overlying layers. It depends on the specific structure of the sample; however, changes as low as 0.5% of the copper concentration produce noticeable changes in the simulated spectrum, while in the case of silver and gold, due mainly to their lower concentrations with respect to the copper one, changes in the simulated spectrum are detected for concentration variations higher than 5%. In all cases the sensitivity values are comparable or better than the expected error on the concentration estimates due to the error in the atomic parameters involved in the calculation, which can be estimated as ranging from 2% to 10%. Thus, even from this aspect the method appears to be high-performing.

## 4. Conclusions

In this paper a new technique was developed and applied for the study of several types of multi-layered metals, from gilding to depleted ones. For the first time a completely non-destructive approach, based on the combination of energy-dispersive X-ray Fluorescence and Monte Carlo simulations was able to discriminate with certainty Tumbaga from gilded copper and single layer Au-based alloys. The same approach, in principle can be easily applied to other types of alloys, such as bronzes. The protocol shows how the elemental concentration varies vs. depth of the multi-layered samples; moreover, it is highly sensitive to variations of the gradient slope as well as changes in the relative concentrations. In conclusion, the developed protocol appears to be a promising and innovative tool for the characterization of multi-layered metals. In the future, we would like to explore the effectiveness of the approach proposed in a controlled situation, that is, with artificial Tumbaga, which allows destructive testing and so comparison between the estimated structure and the real one, obtained by a metallographic exam of the real sample.

## Figures and Tables

**Table 1:** List of samples examined. Each sample is indentified by “S/T1-0:” followed by a number.

Samples (Code :S/T1-O:)	Typology
Chin protector (O:7)	Tumbaga
Nose decoration (O:9)	Tumbaga
Brain container (O:11)	Tumbaga
Right eye protector (O:18)	Tumbaga
Nose protector (O:13)	Tumbaga
center eye protector (=:12)	Tumbaga
Tooth protector (O:23)	Tumbaga
Convex nose protector (O:14)	Au- alloy

**Table 2:** Chin protector structure: and composition of each layer. The realtive concentration are in wt%.

Layers	Gold (Au wt%)	Silver (Ag wt%)	Copper (Cu wt%)	Thickness (μm)
1	61.0	31.4	2.0	0.35
2	60.8	30.7	5.3	0.35
3	59.5	28.9	8.2	0.35
4	59.0	27.0	13.8	0.35
5	58.0	24.4	17.5	0.35
6	57.0	21.9	21.0	0.35
7	56.0	19.5	24.5	0.35
8	53.0	15.7	31.3	0.35
9	50.0	12.0	38.0	0.7
10	47.0	7.0	46.0	0.7
11	40.4	4.5	55.1	bulk

## Data Availability

The data generated is available upon request to the authors.
